# Habituation deficit of visual evoked potentials in migraine patients with hypermobile Ehlers-Danlos syndrome

**DOI:** 10.3389/fneur.2023.1072785

**Published:** 2023-03-09

**Authors:** Ilaria Maestrini, Lorenzo Rocchi, Francesca Puledda, Alessandro Viganò, Giada Giuliani, Tommaso Benedetto Jannini, Claudia Celletti, Marta Altieri, Filippo Camerota, Massimiliano Toscano, Vittorio Di Piero

**Affiliations:** ^1^Department of Human Neurosciences, Headache Centre, “Sapienza” University of Rome, Rome, Italy; ^2^Department of Systems Medicine, University of Rome Tor Vergata, Rome, Italy; ^3^Department of Medical Sciences and Public Health, University of Cagliari, Cagliari, Italy; ^4^Headache Group, Wolfson Centre for Age-Related Diseases (CARD), Institute of Psychiatry, Psychology, and Neuroscience, King's College London, London, United Kingdom; ^5^IRCCS Fondazione Don Carlo Gnocchi, Milan, Italy; ^6^Physical Medicine and Rehabilitation Division, Umberto I Hospital, Rome, Italy; ^7^Department of Neurology, Fatebenefratelli Hospital - Gemelli Isola, Rome, Italy; ^8^University Consortium for Adaptive Disorders and Head Pain (UCADH), Pavia, Italy

**Keywords:** Ehlers-Danlos syndrome, migraine, neurophysiology, visual evoked potential (VEP), habituation deficit

## Abstract

**Objectives:**

Migraine is one of the most frequent clinical manifestations of hypermobile Ehlers-Danlos syndrome (hEDS). The comorbidity between these two diseases has been only partially investigated. We aimed to observe whether neurophysiological alterations described in migraineurs in visual evoked potentials (VEPs) were present in hEDS patients with migraine.

**Methods:**

We enrolled 22 hEDS patients with migraine (hEDS) and 22 non-hEDS patients with migraine (MIG), with and without aura (according to ICHD-3), as well as 22 healthy controls (HC). Repetitive pattern reversal (PR)-VEPs were recorded in basal conditions in all participants. During uninterrupted stimulation, 250 cortical responses were recorded (4,000 Hz sample rate) and divided into epochs of 300 ms after the stimulus. Cerebral responses were divided into five blocks. The habituation was calculated as the slope interpolating the amplitudes in each block, for both the N75-P100 and P100-N145 components of PR-VEP.

**Results:**

We observed a significant habituation deficit of the P100-N145 component of PR-VEP in hEDS compared to HC (*p* = 0.002), unexpectedly more pronounced than in MIG. We observed only a slight habituation deficit of N75-P100 in hEDS, with a slope degree that was intermediate between MIG and HC.

**Discussion:**

hEDS patients with migraine presented an interictal habituation deficit of both VEPs components like MIG. Pathophysiological aspects underlying the pathology could account for the peculiar pattern of habituation in hEDS patients with migraine characterized by a pronounced habituation deficit in the P100-N145 component and a less clear-cut habituation deficit in the N75-P100 component with respect to MIG.

## Introduction

Hypermobile Ehlers-Danlos syndrome (hEDS) is a hereditary disorder of connective tissue consisting of generalized joint hypermobility, connective tissue abnormalities, and musculoskeletal manifestations. It was previously known as EDS type II according to the Berlin nosology and hEDS according to Villefranche nosology (1) and the new International Classification of Ehlers-Danlos Syndromes and Related Disorders (2).

Fatigue and chronic pain are the most frequent neurological symptoms in hEDS (3, 4), with headache being one of the most common and disabling form of pain (5, 6), particularly migraine type (5). In a case-control study, the prevalence of migraine was reported to be 75% in patients with joint hypermobility syndrome (JHS), which is considered a continuum with hEDS because of their indistinguishable clinical features, vs. 43% in healthy controls (5). We recently observed that migraine is more severe in hEDS patients than in normal migraineurs, the former group showing earlier-onset disease, more frequent and disabling attacks, and more severe accompanying symptoms (6). The fact that hEDS patients with migraine are usually undertreated and subsequently at higher risk of chronicization further worsen the migraine burden of these patients. Despite this, the comorbidity between these two disorders has been only partially investigated (6).

Various studies have found significant changes of bioelectrical activity in the visual cortex of migraineurs over the migraine cycle, explored using cortical visual evoked potentials (VEPs) (7, 8). Though not all (9, 10), most studies have shown interictal impaired habituation of VEPs to repeated monotonous stimuli in episodic migraineurs (EM) with and without aura, and an ictal normalization (7, 8). Therefore, impaired habituation has been widely accepted as a biomarker of the interictal status in patients with migraine (11). To the best of our knowledge, there are no investigations on the VEPs pattern in hEDS patients with migraine thus far. From a pathophysiological point of view, different mechanisms have been evoked as possible contributors for headache in hEDS patients: (i) a possible result of ligament laxity, atlantoaxial instability, craniocervical instability, or a separate entity (12), and (ii) a potential association with idiopathic intracranial hypertension (13). Thus, the aim of our study was to address if the aforementioned heavier burden of headache in hEDS patients is due to different neurophysiological aspects, or rather migraine in hEDS patients shares the same pathophysiology of migraineurs without hEDS. Therefore, we tested whether the deficit in VEPs habituation, classically described in patients with migraine, is also observed in hEDS patients with migraine, with the hypothesis that migraine in the two groups shares the same pathophysiology.

## Materials and methods

### Subjects

hEDS patients with migraine (hEDS), with or without aura (ICHD-3 codes 1.2 and 1.1, respectively), were selected among those attending the outpatient multidisciplinary clinic for inherited connective tissue disorders of Physical Medicine and Rehabilitation Division, Umberto I Hospital of Rome. All subjects had normal or normal corrected visual acuity. Prophylactic migraine treatment, neuromodulators, and anti-depressants were not allowed for at least three months before trial inclusion. Migraine was diagnosed by experienced neurologists according to ICHD-3 criteria (14). The diagnosis of hEDS relied on the previous Villefranche and Brighton criteria (1), given that patients were admitted into the outpatient service before the publication of the new classification of EDS and related disorders. All the diagnoses, however, were retrospectively confirmed according to the new criteria for hEDS (2). Consecutive non-hEDS patients with migraine (MIG), with or without aura, matched for monthly headache days and monthly drug intake (number of pills per month), were enrolled at the Headache Clinic of the University Hospital of Rome. According to ICHD3 code 1.3, migraine patients were classified into EM, chronic subgroup (CM), and medication overuse headache (MOH).

The matching between groups was performed by the study coordinator [MT], who was blinded to patients' information except those used for matching. Patients were paired by using a custom-made approach in which each patient of the case group was paired with the first control patient having the same value (±1) in the variables of interest (CM and MOH). In the second permutation, each case-patient was paired with the second patient in the control list having the same characteristics. About ten permutations filled all case-patients and the best one in terms of similar average and standard deviation was chosen. An independent neurologist [IM], blinded for group allocation accurately collected clinical data from patients' clinical records that were used to perform group comparison.

Prophylactic migraine treatment and anti-depressants were not allowed for at least three months before trial inclusion. To avoid bias by hormonal effects, females were recorded at mid-cycle. The neurophysiological recordings were performed during the interictal period (i.e., at least 3 days after and before the last attack) for EM patients and at least 12 h after the intake of symptomatic pain therapy.

We recruited for comparative electrophysiological recordings healthy controls (HC) that were gender- and age-matched to patients and did not have personal or family history of migraine, neurological or psychiatric disease, and did not use drugs for at least 3 months before the recordings.

### Standard protocol approvals, registrations, and patient consent

All subjects provided their written informed consent to participate in this study. This study was approved by the Ethics Committee of Human Experimentation of Policlinico Umberto I University Hospital (Prot n. 152/18 CE ref. 4839) and conformed to the latest version of the declaration of Helsinki.

### Availability of data

The datasets used and/or analyzed during the current study are available from the corresponding author upon reasonable request.

### Pattern-reversal visual evoked potentials (PR-VEPs) recording and processing

PR-VEPs were performed according to current recommendations (15), and similar to the protocol used in our previous studies (16, 17). Briefly, stimuli were presented as a checkerboard pattern of white and black squares (contrast 80%) subtending 1 deg, 8 min of arc, and reversed at a rate of 3.1/s. With one eye patched, subjects were instructed to fixate a colored dot in the middle of the screen of a dimly lit room. Surface electrodes were attached to Oz (active electrode) and Fz (reference) according to the 10/20 system. The ground electrode was placed on the dorsum of the hand. During uninterrupted delivery of 250 stimuli, five blocks of 50 responses were sequentially averaged. We identified N75 as the most negative wave occurring around 75 ms from the stimulus (range 60–90 ms), P100 as the most positive wave occurring after N75 at around 100 ms (range 80–120 ms), N145 as a negative wave around 145 ms (between 125 and 150 ms). We measured the latency of each component (N75, P100, N145) and peak-to-peak amplitude of the N75-P100 and P100-N145 components in each block. The habituation was calculated as the slope interpolating the amplitudes in each block (habituation slope), for both the N75-P100 and P100-N145 components. Negative values of the slope reflect habituation (i.e., a decrement of responses over time), whereas values close to zero or positive indicate a lack of habituation.

### Statistical analysis

All data were analyzed using Statistical Package for the Social Sciences 25 for Windows (IBM Corp, Armonk, USA). A one-way between-group analysis of variance (ANOVA) was used to compare age between the three groups tested (hEDS, MIG, HC). The same test was used to disclose possible differences between hEDS and MIG in terms of monthly headache days. Several Fisher exact tests were performed to investigate possible differences in gender between the three groups, migraine type (episodic, chronic), medication overuse, as well as the presence of aura between the two migraineurs' groups. Several one-way between-group ANOVAs were employed to disclose a possible difference in VEP latency of N75, P100, and N145 in the three groups (hEDS, MIG, HC). Two one-way between-group ANOVAs were performed to assess first block differences (block I) in the three groups regarding N75-P100 and P100-N145 amplitudes. As a synthetic index of within-group habituation, we compared VEP amplitude, separately for N75-P100 and P100-N145 components, between block I and block V using paired t-tests, with the assumption that successful habituation would imply a significant reduction of VEP amplitude in block V, compared to baseline. To assess between-group differences in habituation, we modeled the amplitude of VEP with a straight line and calculated its slope at the subject level for each group (hEDS, MIG, HC) and VEP components (N75-P100 and P100-N145); the slope values obtained in this way were entered in two one-way between-group ANOVAs, one for each VEP component. Normality of distribution with the Shapiro-Wilks' test. Levene's test was used to investigate possible inhomogeneities of variance across groups. To test for data sphericity, we used Mauchly's test; when sphericity was violated (i.e., Mauchly's test < 0.05), we used the Greenhouse-Geisser correction. Pairwise comparisons were corrected by the Bonferroni method. *P* values < 0.05 were deemed significant.

## Results

We enrolled twenty-two patients for each group (hEDS, MIG, and HC). The clinical features of participants in the three groups are reported in Table 1.

**Table 1:** Clinical and demographic features of the three groups.

	**hEDS *n* = 22**	**MIG *n* = 22**	**HC *n* = 22**	**Statistics values**	***p*-values**
Age (years)	29.32 ± 12.30^+^	30.64 ± 10.34^+^	32.91 ± 6.48^+^	F_2.63_ = 1.189	0.311
Gender	18F, 4M	19F, 3M	16F, 6M	χ^2^ (2) = 1.341	0.511
Type of migraine	9EM, 13CM	9EM, 13CM	–	χ^2^ (1) = 0.727	0.394
Migraine with aura	10	1	–	χ^2^ (1) = 18.182	**<0.001**
Monthly headache days	15.91 ± 11.57^+^	15.00 ± 11.63^+^	–	–	0.796
Medication overuse	6	8	–	χ^2^ (2) = 0.419	0.747

Unless specified, values are the number of patients. ^+^ indicates Mean ± standard deviation. Bold values indicate statistical significance at the *p* < 0.05 level.

Age was homogeneous among the three groups. hEDS and MIG patients did not differ in terms of monthly headache days. There were no gender differences between groups. As a result of matching, both the rate of chronic migraine (CM) and medication overuse headache (MOH) was similar between hEDS and MIG groups (*p*-values 0.394 and 0.747, respectively). The former group showed a higher prevalence of aura, compared with the latter (*p* < 0.001). All patients who had migraine with aura presented a visual aura and all patients with MOH overused nonsteroidal anti-inflammatory drugs (NSAIDs) or mixed drugs (NSAIDs and triptans).

The grand average PR-VEPs across subjects in the three groups (i.e., hEDS, MIG and HC) is shown in Supplementary Figure 1.

**Figure 1:**
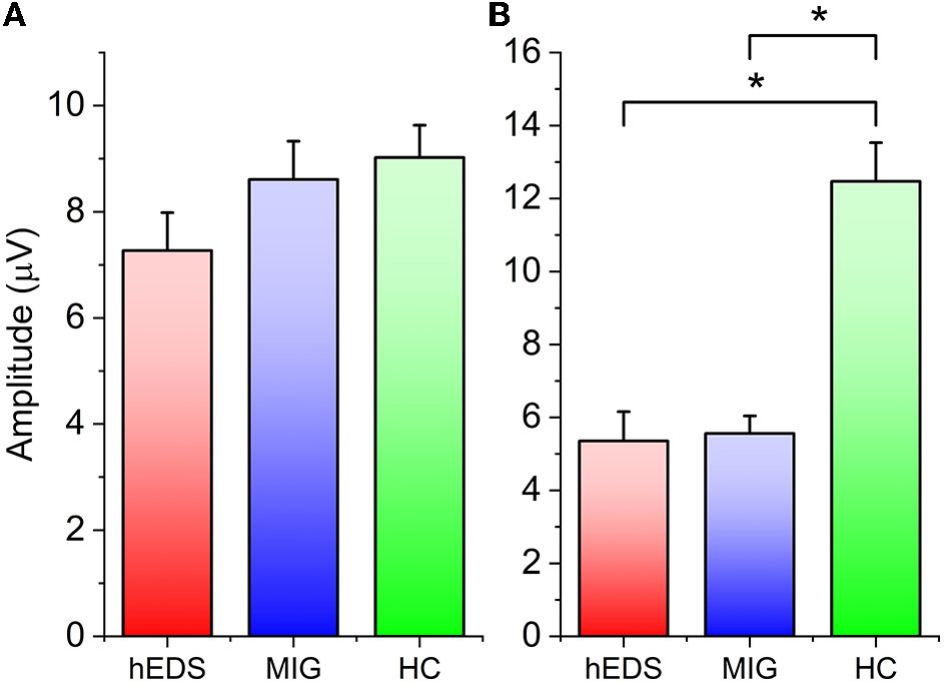
**(A)** N75-P100 baseline (block I) amplitude. **(B)** P100-N145 baseline (block I) amplitude. Asterisks indicate statistically significant difference (*p* value < 0.05).

VEP latencies were not significantly different across the three groups (Table 2). The amplitude of N75-P100 did not differ across groups, whereas the P100-N145 first block amplitude was lower in hEDS and MIG than HC (*p* = 0.001 and *p* < 0.001, respectively) (Table 2 and Figure 1). The within-group comparisons between N75-P100 amplitudes in blocks I and V did not disclose any significant p values in patients; by contrast, HC showed a different trend, with the amplitude of block V being significantly smaller than the block I, thus indicating habituation (t_22_ = 4.255, *p* < 0.001).

**Table 2:** Comparison of VEP components by one-way ANOVAs with factor group (hEDS and MIG patients with migraine, and healthy controls) and *post-hoc* analysis.

	**hEDS**	**MIG**	**HC**	**F statistics**	**ANOVA *p*-values**	**hEDS vs HC**	**MIG vs HC**	**hEDS vs MIG**
**VEP latencies (ms)**								
N75	78.59 ± 6.10	81.09 ± 3.31	79.77 ± 5.52	F_2, 63_ = 1.313	0.275	0.999	0.998	0.331
P100	115.45 ± 5.83	116.55 ± 5.13	112.95 ± 5.54	F_2, 63_ = 2.457	0.094	0.412	0.103	0.974
N145	157.55 ± 11.18	160 ± 14.71	153.82 ± 12.75	F_2, 63_ = 1.269	0.288	0.968	0.356	0.969
**N75-P100**								
Amplitude (μV)	7.27 ± 3.35	8.61 ± 3.38	9.02 ± 0.61	F_2, 63_ = 1.806	0.173	0.222	0.988	0.509
Slope	−0.12 ± 0.11	0.34 ± 0.16	−0.18 ± 0.17	F_2, 63_ = 6.198	**0.003**	0.991	**0.004**	**0.029**
**P100-N145**								
Amplitude (μV)	5.35 ± 3.75	5.56 ± 2.24	12.47 ± 1.06	F_2, 63_ = 24.569	**< 0.001**	**0.001**	**< 0.001**	0.996
Slope	0.53 ± 0.20	0.40 ± 0.19	−0.20 ± 0.07	F_2, 63_ = 7.627	**0.001**	**0.002**	**0.009**	0.984

Comparative group-to-group significant (<0.05) values are in bold.

**Figure 2:**
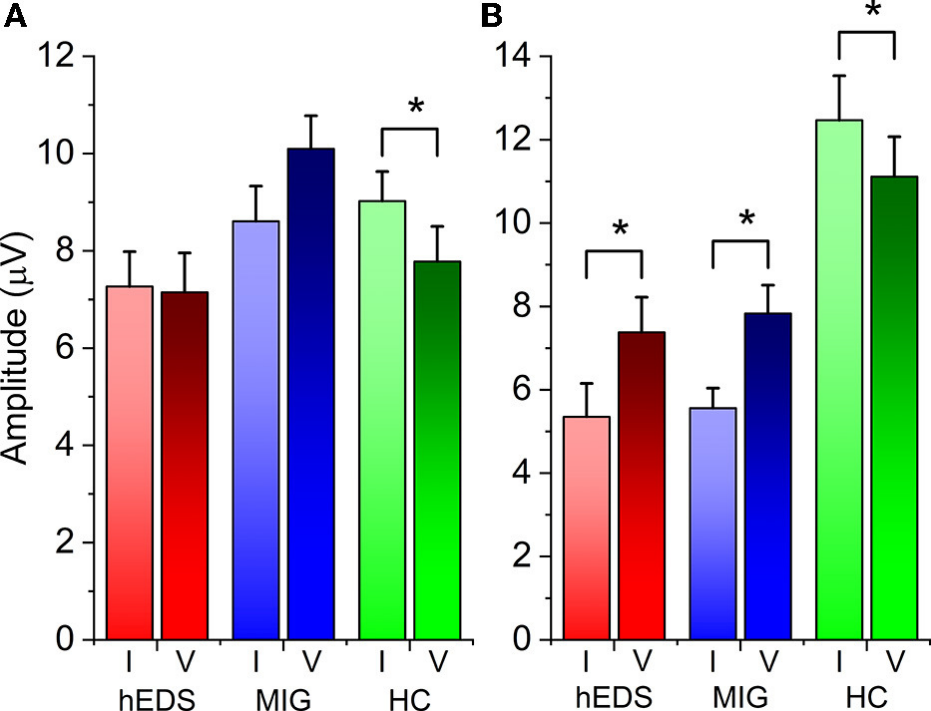
**(A)** Within-group habituation: block I vs block V N75-P100. **(B)** Within-group habituation: block I vs. block V P100-N145. Asterisks indicate statistically significant difference (*p* value < 0.05).

The amplitude of P100-N145 was higher in block V than block I in both hEDS (*p* = 0.018) and MIG (*p* = 0.006), whereas the same comparison showed a significant decrease in HC, again pointing toward successful habituation in the last group (*t*_22_ = 2.295, *p* = 0.032) (Figure 2).

**Figure 3:**
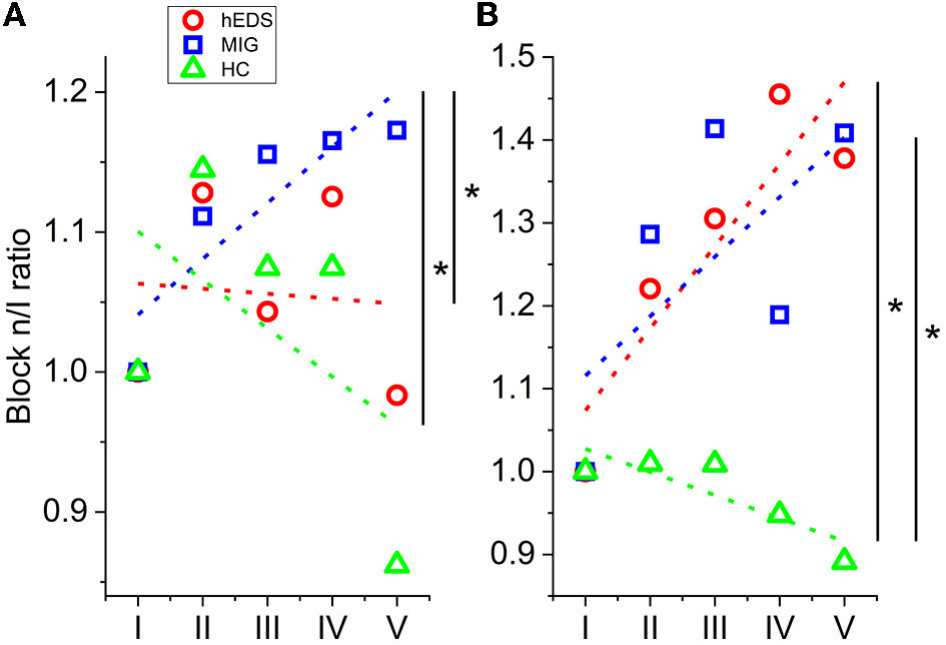
**(A)** N75-P100 slope. **(B)** P100-N145 slope. Asterisks indicate statistically significant difference (*p* value < 0.05).

The amplitude slope values for the N75-P100 VEP components were significantly higher for MIG than HC (*p* = 0.004) and for MIG than hEDS (*p* = 0.029). Additionally, when considering the P100-N145 component, amplitude slope values were higher for both hEDS and MIG, compared to HC (*p*-values 0.004 and 0.015, respectively) (Figure 3).

### Additional analyses

The described results raised two further questions. The first is whether the habituation of the P100-N145 component of VEP was driven by the first-block amplitude value of the same component, which was smaller in patients than in HC. To address this hypothesis, we performed a between-group analysis of covariance (ANCOVA) on the slope of P100-N145 VEP, using block I P100-N145 amplitude as a covariate. This analysis showed a significant effect of “group” (F2,63 = 4.726, *p* = 0.01). *Post hoc* comparisons confirmed a higher slope in hEDS and MIG, compared to HC (*p*-values 0.014 and 0.029, respectively). This means that, despite factoring out the effect of different baseline amplitude of P100-N145, the differences highlighted by the previous ANOVA are valid.

Another question pertains to the difference between the two groups of patients in terms of the slope of N75-P100 VEP components. Since the only clinical difference between the two groups was in the prevalence of aura, we verified whether this factor could have driven the difference in the N75-P100 slope by performing a between-group ANCOVA with the presence of aura as a covariate. When factoring out the effect of the aura, the ANCOVA still shows a significant effect of factor “group” (F2,63 = 6.275, p = 0.03), supporting the notion that the presence of aura does not affect the result.

## Discussion

Our study yielded three main results: (i) as expected, MIG showed a significant PR-VEP habituation deficit in both N75-P100 and P100-N145 components, compared to HC; (ii) hEDS showed a more pronounced PR-VEP habituation deficit in the P100-N145 component than MIG, while only a slight deficit of habituation of N75-P100, with a slope degree that was intermediate between MIG and HC; (iii) both MIG and hEDS groups showed a reduction in baseline P100-N145 component amplitude compared to HC.

### Interictal VEP habituation deficit in MIG

Consistent with previous studies (11), we found impaired habituation of N75-P100 and P100-N145 components of PR-VEPs among MIG patients. In our hEDS cohort, due to the low prevalence of the disease, we enrolled patients suffering from both EM and CM, and those with MOH as well. Then, we matched hEDS and MIG groups according to the presence of CM and MOH. The rationale behind this matching is that habituation differs based on migraine frequency, being normal in chronic phenotype, similar to ictal EM recordings so that CM has been defined as a “never-ending attack” (18–20). Moreover, patients with MOH show a different electrophysiological pattern from that underlying EM (i.e., initial amplitude increase of VEP, with subsequent lack of habituation) (21). Importantly, despite a mixed cohort, in our study, both groups presented impaired habituation, probably driven by the EM phenotype.

Regarding other possible factors that may have influenced the habituation deficit, the two groups of patients with migraine (hEDS and MIG) significantly differed in terms of aura prevalence. We decided not to match the two groups for the presence of aura because patients suffering from migraine with aura present an interictal habituation deficit like those with migraine without aura, even though this datum is less consistent in literature (22). Anyway, the visual cortex hyperactivity has been implicated in etiological mechanisms of cortical spreading depression (23), which is the proposed neurophysiological correlate of migraine aura. Especially V3A (a subregion of the extrastriate visual cortex) has been considered a potential source of cortical spreading depression in patients with migraine with visual aura (24).

To address this possible inclusion bias, we performed an additional between-group ANCOVA with the presence of aura as a covariate, and the “group” effect remains significant even after the exclusion of the aura effect. A bias from comorbid migraine with aura is therefore unlikely. Moreover, a recent review on electrophysiological patterns in migraine with aura patients described inconsistent findings of N75-P100 and/or P100-N145 VEPs amplitudes, that were reported to be greater or reduced or, most often, in the normal range compared to migraine without aura and controls (22).

### Habituation pattern in hEDS

hEDS showed an interictal habituation deficit of both components of VEPs, similar to MIG. This result confirms, from a neurophysiological point of view, the clinical finding of our previous study that migraine in hEDS represents a separate entity, even in the context of a syndrome characterized mainly by generalized pain (6).

Anyway, hEDS patients suffered from a more severe phenotype of migraine showing earlier-onset, higher frequency, more severe accompanying symptoms, and higher scores on disability assessment questionnaires with respect to patients with isolated EM (6). Therefore, we hypothesized that in these patients the pathophysiological aspects peculiar to hEDS were added to migraine (6).

In this regard, it is worth noting that, in the present study, hEDS patients with migraine showed a more pronounced PR-VEP habituation deficit in the P100-N145 component than in the N75-P100 one. Classically, a visual stimulus is initially processed in the striate cortex, and subsequently, each primary visual area transmits the information in two main directions, detectable as an N145 wave in the VEPs (24). Thus, the cortical generator of the two components is different and represented by striatal areas (Brodmann area 17) for the N75-P100 component, and extrastriate areas (Brodmann area 18 and 19) for the P100-N145 component (25, 26). In particular, P100-N145 potentials are generated by the transit of information from the primary to the secondary and associative cortices and are known as ventral current (*ventral stream)* and dorsal current (*dorsal stream*) (25). The dorsal visual network (DVN) area is adjacent to the parietal lobe in the dorsal stream, which stretches from the primary visual area into the parietal lobe and is associated with spatial awareness and guidance of actions.

Thus, given the network subtended to the P100-N145 component of VEPs, we hypothesize that the pronounced habituation deficit of this component observed in the hEDS may be related to functional alterations of extrastriate cortical network, like the DVN, that are crucially involved in the chronic pain experienced by these patients. In this perspective, the DVN participates in the cognitive selection of relevant sensory information and can enhance visual attention by determining the cognitive integration of different sensory modalities within the central nervous system, again with the salience network (previously called “pain matrix”) (27). In a very recent study from Coppola et al. (28), increased neural connectivity between the hypothalamus and brain areas belonging to the default mode network and DVN has been detected in a population of twenty patients with CM without medication overuse who underwent 3-T MRI scans using a 7.5-min resting-state protocol. Authors concluded that the hyper-connectivity of the hypothalamus with the default mode network and the DVN might be an adaptive, and presumably ineffective, coping strategy to enhance avoidance learning for events associated with stressful negative outcomes such as persistent chronic headache. In this way, the lack of physiological mechanisms to select relevant information could interfere with the cognitive attenuation of pain perception due to the low strength of the functional connectivity between the hypothalamus and the medial prefrontal cortex participating in pain chronicization (28).

Remarkably, the same correlation between the medial prefrontal cortex and severity of pain has been found in other chronic painful conditions such as chronic low back pain (29) and fibromyalgia (30).

To our knowledge, there are no further electrophysiological studies investigating habituation in VEPs in other chronic pain syndromes, but few studies on habituation of different sensory modalities could support our hypothesis (31–35).

However, it should be noted that hEDS showed only a slight deficit of habituation of N75-P100, with a slope degree that was intermediate between MIG and HC.

The lack of a clear-cut habituation deficit in the N75-P100 component of VEPs in hEDS compared to MIG, suggests that perhaps more than one pathophysiological mechanism has a role in the complex migraine phenotype of hEDS. In a single-center retrospective study on one-hundred and forty patients with hypermobility disorders, 66% reported either headache -mostly migraine- or cervicalgia, mainly cervical spondylosis, coexisting in almost more than half of the population over a 2-year period (36).

To our knowledge, no studies are available on neurophysiological patterns of cervicogenic headache, so further studies are warranted to elucidate this point. In our population, we did not specifically investigate the coexistence of cervicalgia or spinal pathology. Moreover, the study's main limitations rely on the small sample size and the absence of a control population of hEDS patients without migraine, due to the rarity of the disease. Thus, we could only hypothesize that this peculiar neurophysiological pattern is partially related to a possible cervicogenic trigger that might interfere with the habituation deficit of the N75-P100 component of VEPs or other disorders intrinsic to hEDS (i.e., connective tissue disorders) that might have influenced VEPs. Moreover, we could not exclude a common genetic basis underlying hEDS and migraine, even though the genetic basis of the hEDS is still unknown (12), and the probability is accordingly difficult to estimate.

Albeit there is evidence that the N75-P100 and the P100-N145 components of VEPs are related to distinct cortical generators (25, 26), the fact that we used a single recording channel limits possible inferences about differential activation of brain areas.

### Amplitude in the P100-N145 component of PR-VEPs

The habituation deficit of patients with EM is usually accompanied by a normal to decreased amplitude of early responses in averaged data, whereas several studies were not able to reproduce the same results, probably because of the variation of the habituation impairment over the migraine cycle (11).

Our study confirmed a reduction in baseline amplitude of the P100-N145 component of PR-VEPs in patients with migraine (i.e., both hEDS and MIG). To avoid a possible bias, we investigated the confounding effect of baseline P100-N145 amplitude used as a covariate in an additional between-group ANCOVA on the slope of P100-N145 VEP and found that the habituation of the P100-N145 component was not driven by the reduced baseline amplitude.

Moreover, the amplitude of P100-N145 was significantly higher in block V than block I in both hEDS and MIG compared to HC, as further proof of habituation impairment in both migraineurs' groups. Therefore, these data further support our hypothesis that the more pronounced habituation deficit in the P100-N145 component of PR-VEPs observed in hEDS compared to MIG, could be explained by functional alterations of extrastriate cortical areas, as well as the DVN, that is involved in the chronic pain experienced by these patients due to the underlying disease.

The specific underlying causes and mechanisms of pain in hEDS remain poorly understood. Many factors may contribute to the generation and chronicity of pain: (i) nociceptive pain, directly related to structural changes in affected joints, muscle, and connective tissue, (ii) neuropathic pain, (iii) impaired proprioception and muscle weakness, and (iv) central sensitization. These mechanisms are not mutually exclusive, and likely more than one mechanism may be present. Furthermore, comorbid anxiety and depression, as well as other variables may influence the phenotype of presentation (37). Functional neuroimaging studies are needed to confirm this hypothesis.

hEDS is a disabling, rare, and probably underdiagnosed disease with no specific therapeutic options (38). These results provide insight into the mechanisms underpinning the coexistence of overlapping pain syndromes in hEDS patients and could inform on personalized pharmacological and rehabilitative treatments.

## Data availability statement

The datasets used and/or analyzed during the current study are available from the corresponding author upon reasonable request.

## Ethics statement

The studies involving human participants were reviewed and approved by Ethics Committee of Human Experimentation of Policlinico Umberto I University Hospital. The patients/participants provided their written informed consent to participate in this study.

## Author contributions

IM and MT wrote the main manuscript text. LR performed statistical analyses and prepared Figures 1–3. IM, MT, FP, and AV contributed substantially to the study's conception and design. Material preparation and data collection were performed by IM, MT, FP, AV, and GG. The matching between study's groups was performed by MT, who was blinded to patients' information except those used for matching. IM blinded for group allocation accurately collected clinical data from patients' clinical records. All authors made a substantial contribution to data interpretation and reviewed the manuscript. All authors have read and approved the final manuscript.
