# Supplementary figures and images for: Habituation deficit of visual evoked potentials in migraine patients with hypermobile Ehlers-Danlos syndrome
Source: Front Neurol. 2023 Mar 9;14:1072785. doi: 10.3389/fneur.2023.1072785 (PMC10034036; doi:10.3389/fneur.2023.1072785)

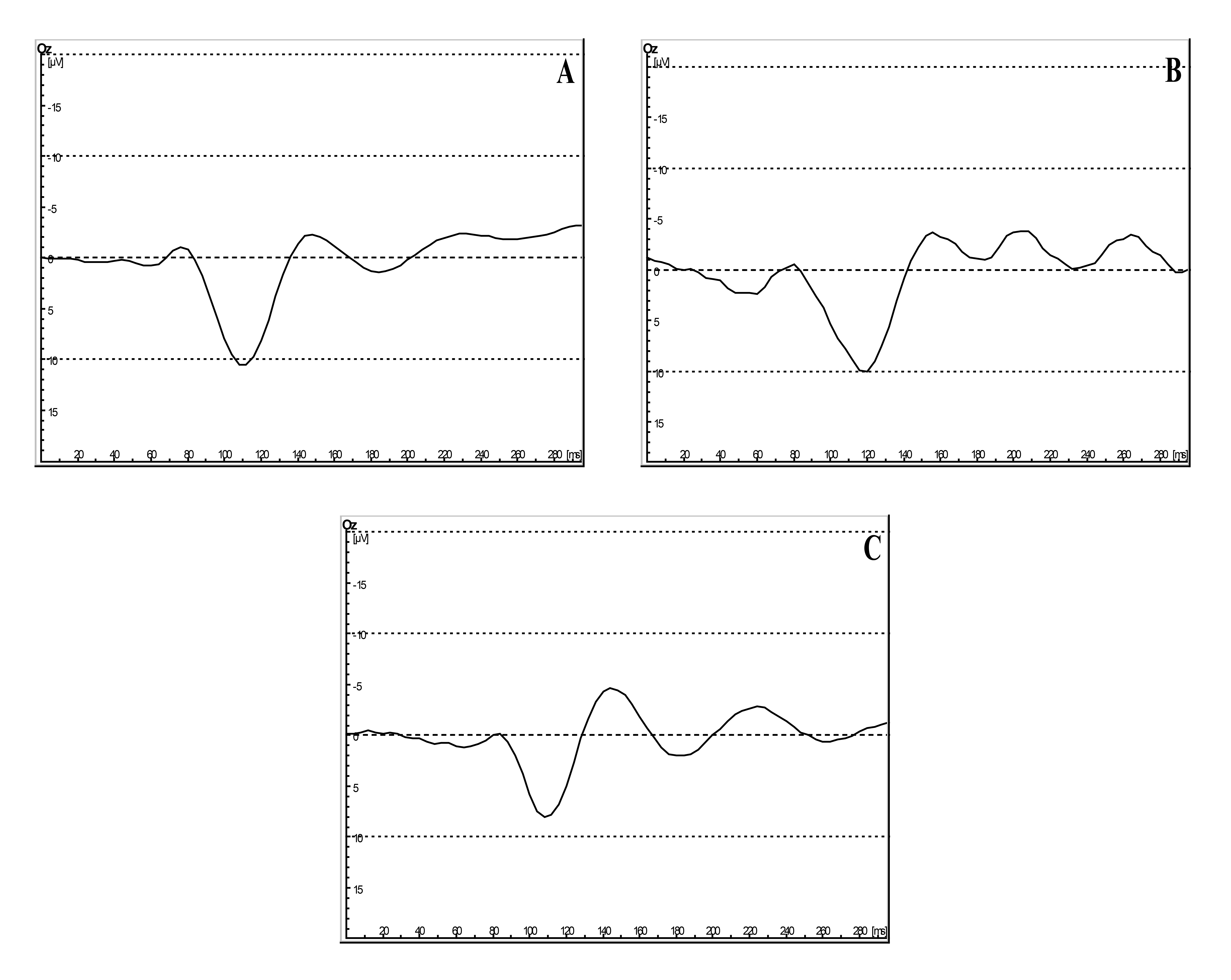

Supplement: Supplementary file 1 [file Image_1.jpg]
